# Inactivation of BoORP3a, an oxysterol-binding protein, causes a low wax phenotype in ornamental kale

**DOI:** 10.1093/hr/uhac219

**Published:** 2022-09-28

**Authors:** Simeng Zhang, Fuhui Zhou, Zheng Liu, Xin Feng, Yashu Li, Pengfang Zhu

**Affiliations:** College of Forestry, Shenyang Agricultural University, Shenyang, 110866, China; Key Laboratory of Forest Tree Genetics, Breeding and Cultivation of Liaoning Province, Shenyang, 110866, China; College of Forestry, Shenyang Agricultural University, Shenyang, 110866, China; Key Laboratory of Forest Tree Genetics, Breeding and Cultivation of Liaoning Province, Shenyang, 110866, China; College of Forestry, Shenyang Agricultural University, Shenyang, 110866, China; Key Laboratory of Forest Tree Genetics, Breeding and Cultivation of Liaoning Province, Shenyang, 110866, China; College of Forestry, Shenyang Agricultural University, Shenyang, 110866, China; Key Laboratory of Forest Tree Genetics, Breeding and Cultivation of Liaoning Province, Shenyang, 110866, China; College of Forestry, Shenyang Agricultural University, Shenyang, 110866, China; Key Laboratory of Forest Tree Genetics, Breeding and Cultivation of Liaoning Province, Shenyang, 110866, China; College of Forestry, Shenyang Agricultural University, Shenyang, 110866, China; Key Laboratory of Forest Tree Genetics, Breeding and Cultivation of Liaoning Province, Shenyang, 110866, China

## Abstract

Identifying genes associated with wax deposition may contribute to the genetic improvement of ornamental kale. Here, we characterized a candidate gene for wax contents, *BoORP3a*, encoding an oxysterol-binding protein. We sequenced the *BoORP3a* gene and coding sequence from the high-wax line S0835 and the low-wax line F0819, which revealed 12 single nucleotide polymorphisms between the two lines, of which six caused five amino acids substitutions. BoORP3a appeared to be relatively well conserved in Brassicaceae, as determined by a phylogenetic analysis, and localized to the endoplasmic reticulum and the nucleus. To confirm the role of BoORP3a in wax deposition, we generated three *orp3a* mutants in a high-wax kale background via CRISPR/Cas9-mediated genome editing. Importantly, all three mutants exhibited lower wax contents and glossy leaves. Overall, these data suggest that *BoORP3a* may participate in cuticular wax deposition in ornamental kale.

## Introduction

Wax located outside the cuticle or within the cuticular matrix plays many roles in supporting plant growth and survival in various environments. As a hydrophobic barrier, the cuticular wax primarily reduces transpirational water loss and thus enhances drought tolerance [1]. The cuticular wax also provides a physical barrier that protects plants against microbial infections, insect attacks, and damage from ultraviolet (UV) light. In addition, the cuticular wax layer is waterproof and self-cleaning to prevent the deposition of dust and other pollutants [1, 2]. Cuticular wax has been shown to affect plant development, pigmentation, and fertility [3]. The cuticular wax is composed of very-long-chain fatty acids (VLCFAs) and their derivatives, alcohols, alkanes, alkenes, aldehydes, esters, ketones, triterpenoids, and sterols [4]. The biosynthesis of cuticular wax takes place within epidermal cells [5]. The C16 and C18 fatty acids begin to form in plastids, followed by fatty acid elongation into VLCFAs mediated by the fatty acid elongase complex [6]. VLCFAs are subsequently modified via the alkane- and alcohol-forming pathways [7], while the primary alcohol and wax ester are derived from the alcohol-forming pathway. The aldehydes, alkanes, secondary alcohols, and ketones are produced by the alkane-forming pathway [8]. These wax components are collectively exported to the cuticular wax layer by members of the ATP binding cassette (ABC) transporter and glycosylphosphatidylinositol-anchored lipid transfer protein (LTPG) families [9, 10]. Several genes involved in wax biosynthesis, export, and regulation have been identified in Arabidopsis (*Arabidopsis thaliana*), such as *ECERIFERUM1* (*CER1*)/*CER3*/*CER4–7*/*CER10*, *CURLY FLAG LEAF1* (*CFL1*),*FATTY ACYL-ACP THIOESTERASE B* (*FATB*), *WAX SYNTHASE/ACYL-CoA*:*DIACYLGLYCEROL ACYLTRANSFERASE1* (*WSD1*), *ABCG11*, and*LTPG1* [3]. However, the molecular mechanisms linking these and other genes with cuticular wax transport are poorly understood.

Sterols are vital cellular components that contribute to the maintenance of membrane integrity, metabolism related tomembrane properties, secretory trafficking events, as well as the biosynthesis of wax, cellulose, callose, lignin, and the phytohormone brassinosteroids [4, 11, 12]. Sterol biosynthesis occurs in the endoplasmic reticulum (ER) but later mainly accumulates in theplasma membrane (PM) [13]. Oxysterol-binding proteins (OSBPs) and OSBP-related proteins (ORPs) transport and regulate the metabolism of sterols and phospholipids [14]. ORPs also bind to various lipids, such as ergosterol and cholesterol [13]. Arabidopsis ORP3a, a sterol-binding protein, may participate in the export ofsterols from the ER and their circulation between the ER and the Golgi [13, 15]. Based on the function of ORP3a, we speculate that ORP3a may play a role in the deposition of cuticular wax.

Ornamental kale (*Brassica oleracea var. acephala*) is used not only as a leafy vegetable but also as an ornamental crop, owingto its nutritional value (rich in glucosinolates, phenolic compounds, and carotenoids) and attractive and colorful leaves [16, 17]. The cuticular wax covering ornamental kale organs may enhance plant tolerance to environmental stresses and changes. However, this agronomic trait also significantly influences the edible and ornamental properties of ornamental kale. Indeed,wax was reported to affect fruit postharvest quality and storage capacity [18]. *Brassica* plants covered with cuticular wax areusually glaucous, while wax-free mutant plants typically have a glossy and green color [19]. Thus, exploring how wax forms and is deposited on various organs is critical in ornamental kale to modulate its visual appeal and nutritional value. To date, no study has reported on wax deposition genes in ornamental kale. Our previous study constructed a segregating population between S0835 and F0819 [16]. We exploited bulked-segregant analysis combined with next-generation sequencing (BSA-seq) to identify the candidate gene(s) responsible for the difference in leaf shape and wax contents between these two inbred lines. Based on the functional annotation of genes from the candidate region, we selected *BoORP3a* (*Bo9g184810*), encoding an oxysterol-binding protein, as a high-confidence wax deposition candidate. In the present study, we compared the wax deposition and distribution patterns in two ornamental kale inbred lines with contrasting wax phenotypes by scanning electron microscopy and measured their wax contents by colorimetry. We also cloned the *BoORP3a* candidate gene for wax deposition and determined the subcellular localization of its encoded protein. Finally, we validated the function of BoORP3a in wax deposition by generating genome-edited mutants via clustered regularly interspaced short palindromic repeats (CRISPR)/CRISPR-associated nuclease 9 (Cas9). The results presented here will contribute to our understanding of the functions associated with the ORP family in ornamental kale.

## Results

### Characterization of cuticular wax in two ornamental kale inbred lines

**Figure 1:**
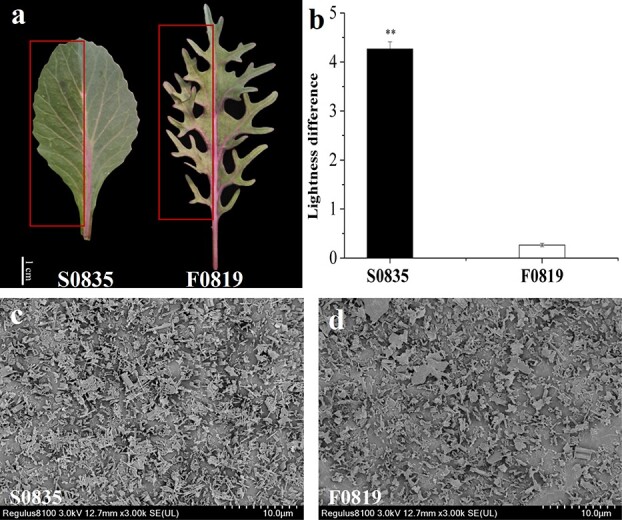
Characterization of cuticular wax in the ornamental kale inbred lines S0835 with high-wax and F0819 with low-wax. **a** Leaf morphology and (**b**) lightness difference of the two inbred lines before and after cuticular wax removal. The red box indicates the part of the leaf with the wax removed. Data are means ± SD (*n* = 5). Asterisks indicate statistically significant differences (*P* < 0.01). **c**, **d** Cuticular wax characterization of the two inbred lines observed by SEM.

We determined the differences in cuticular wax contents and pattern between two ornamental kale inbred lines (Fig. 1a) by scanning electron microscopy (SEM) observations of the leaf surface and quantification of wax contents by colorimetry. We measured the lightness value of leaves before and after wax removal and calculated the lightness difference as an indicator of wax contents (Fig. 1b). Compared to the inbred line S0835, F0819 was characterized by a lower wax content on its leaves (*P* < 0.01). In the S0835 inbred line, the leaf surface was densely and evenly covered with wax crystals, which was in sharp contrast to the leaf surface for F0819, which exhibited sparse wax crystals, in agreement with the lightness difference results (Fig. 1c and d). However, we observed no apparent differences in wax morphology between the two inbred lines. These results indicate that these two inbred lines differ only in their cuticular wax contents, with S0835 having a high wax content.

### Phylogenetic and sequence analysis of *BoORP3a*

In our previous study, a smooth-leaved inbred line (S0835) and a feathered-leaved inbred line (F0819) were used as parental lines to construct the segregating population for BSA-seq and fine mapping [16]. The genetic analysis showed that the feathered-leaved trait was controlled by a semi-dominant gene. Furthermore, we observed the smooth-leaved inbred line always showed more cuticular wax content, while the feathered-leaved inbred line always showed lower cuticular wax content, so it was speculated that these two traits showed linkage. The leaf shape gene was mapped to a 374.532-kb interval and 38 genes were identified, one of them is *Bo9g184810* which encodes an oxysterol-binding protein (ORP3a) [16]. Thus, we speculated the *BoORP3a* gene might confer the cuticular wax content trait.

**Figure 2:**
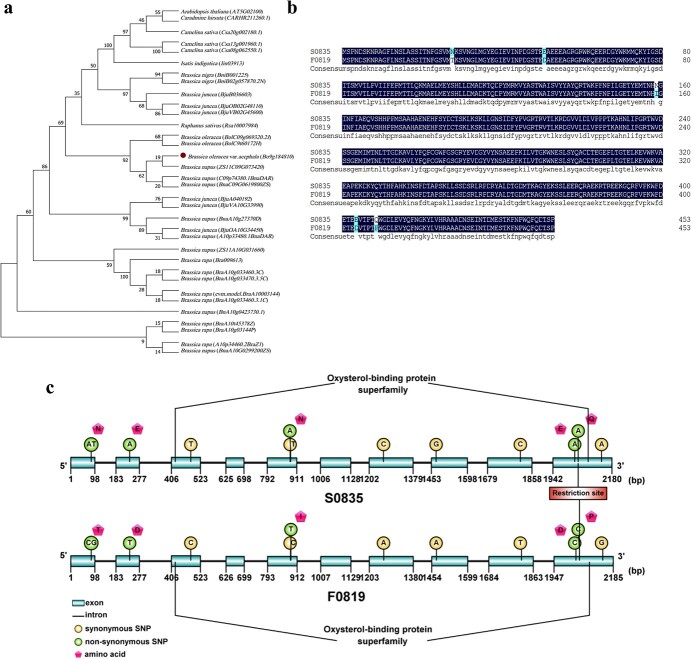
Sequencing analysis of *BoORP3a*. **a** Phylogenetic tree of ORP3a-related proteins across the indicated cruciferous species. **b** Amino acid sequence and (**c**) gene structure analysis of the *BoORP3a* locus in the two inbred lines.

To explore the possible function(s) of BoORP3a, we cloned the encoding gene from the two inbred lines S0835 and F0819. Then, we identified the presence of three homologous sequences of BoORP3a gene within the *B. oleracea* genome (Fig. S2). Only *Bo9g184810* is an orthologue of the *Arabidopsis thaliana ORP3a* gene (*AT5G02100*), and the other three genes are orthologues of the *Arabidopsis thaliana ORP3b* gene (*AT3G09300*). We then used the predicted protein sequence from BoORP3a in S0835 to identify ORP3a-related proteins in 10 cruciferous species (Fig. S3), from which we constructed a phylogenetic tree. The phylogenetic tree indicated that BoORP3a clusters with ORP3a-like proteins from other Brassica species (Fig. 2a). These results suggest that ORP3a is highly conserved in the Brassicaceae. To better understand the evolutionary constraints acting on the ORP3a genes in the Brassicaceae, we calculated the Ka, Ks, and the Ka/Ks ratio. The Ka/Ks ratio of most paralogous gene pairs was <1 (Table S2), indicating that the ORP3a genes experienced purifying selection. Furthermore, 26 pairs with a Ka/Ks value greater than 1 are under positive selection. We aligned the full-length, coding sequence and encoded amino acid sequence of BoORP3a from the two inbred lines. The full-length genomic region (from the translation start site [ATG] to the stop codon) of BoORP3 was 2,180 bp in length in S0835 and 2,185 bp in F0819 (Fig. S4). The BoORP3 coding sequence was of identical length in the two inbred lines at 1,362 bp and encoded a protein of 453 amino acids (Fig. 2b; Fig. S5). The comparison of the two genomic sequences revealed 20 single nucleotide polymorphisms (SNPs) and a 5-bp deletion in S0835 (Fig. S3). The deletion did not affect the coding sequence, but 12 of the 20 SNPs did, of which six caused five amino acid substitutions, N28T, E51D, N159I, E404D, and Q409P from S0835 to F0819 (Fig. S4, Fig. 2b and 2c). The protein encoded by both inbred lines contained a conserved domain belonging to the oxysterol-binding protein superfamily from amino acids 70 to 422 (Fig. 2c). Three of the five amino acid substitutions were within this conserved domain. These results indicate that the polymorphisms at BoORP3a between the inbred lines S0835 and F0819 might affect BoORP3a function and contribute to the change in wax contents.

### Subcellular localization of BoORP3a

**Figure 3:**
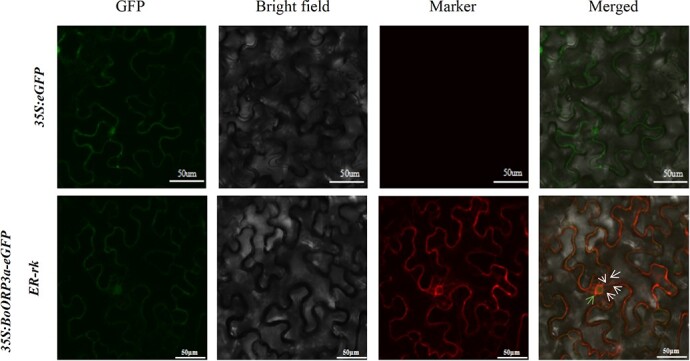
Subcellular localization of BoORP3a. GFP, green fluorescent protein; *35S:eGFP*, *N. benthamiana* leaves expressing *eGFP* alone; *35S:BoORP3a-eGFP*, *N. benthamiana* leaves expressing *BoORP3a-eGFP*. White arrows indicate endoplasmic reticulum; green arrow indicates the nucleus. Scale bars = 50 μm.

We investigated the subcellular location of BoORP3a by transiently infiltrating a construct encoding a fusion between BoORP3a and green fluorescent protein (GFP) into the leaves of *Nicotiana benthamiana* plants, together with a fluorescent marker for the ER. We detected the fusion protein in the ER and the nucleus (Fig. 3).

### CRISPR/Cas9-mediated mutagenesis of *BoORP3a* in ornamental kale

**Figure 4:**
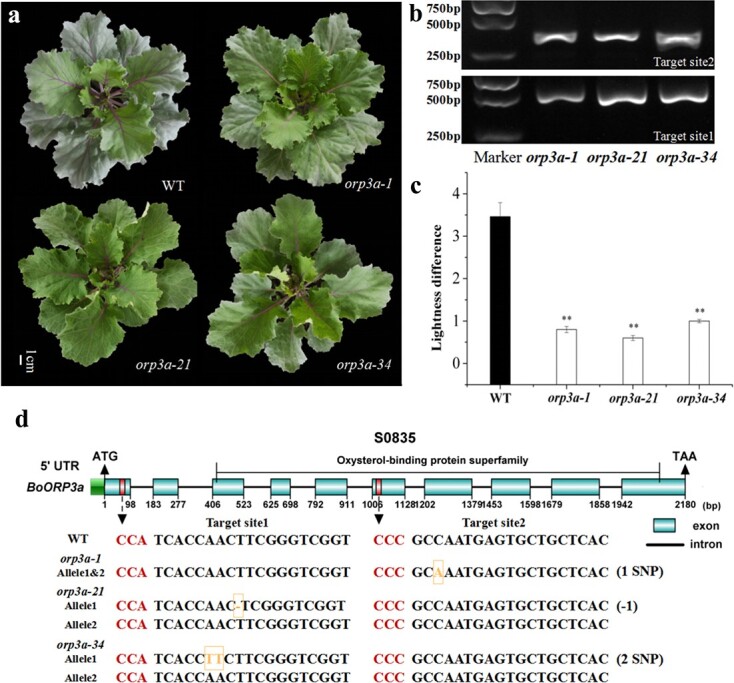
Characterization of CRISPR/Cas9-induced *orp3a* mutants. **a** Representative images of wild-type (WT) and *orp3a* mutant plants. **b** PCR amplification of genomic DNA isolated from *orp3a* mutant plants with primers flanking the sgRNA target sites. **c** Lightness difference in WT and *orp3a* mutant plants. Data are means ± SD (*n* = 5). Asterisks indicate statistically significant differences (*P* < 0.01). **d** Schematic diagram of the *BoORP3a* locus and mutation analysis of the three *orp3a* mutants created by CRISPR/Cas9-mediated genome editing. Substitutions and deletions are highlighted in yellow.

To confirm the function of *BoORP3a* in ornamental kale, we employed CRISPR/Cas9-mediated gene editing to introduce mutations in this gene in the S0835 background (high-wax line). We obtained 40 kanamycin-resistant primary transformant (T_0_) plants, which we then subjected to PCR genotyping and sequencing. To this end, we amplified and sequenced the genomic region covering the target site of the sgRNAs from each transgenic plant (Fig. 4b). We identified three transformants with substitutions and deletion mutations within *BoORP3a* (Fig. 4d). Of the three mutants, only *orp3a-1* was homozygous, with a 1-bp mutation at target site 2, resulting in a P173Q amino acid substitution (Fig. 4d; Fig. S6, see online supplementary material). We detected a 1-bp deletion at target site 1 in *orp3a-21*, resulting in the frameshift mutation after 22 amino acids (Fig. 4d; Fig. S5, see online supplementary material). In *orp3a-34*, we identified two adjacent substitutions at target site 1, resulting in a N22F amino acid substitution (Fig. 4d; Fig. S5, see online supplementary material). As illustrated in Fig. 4a, we observed a marked difference in appearance between the wild-type (WT) and *orp3a* mutant (T_0_) plants. Indeed, all three mutants had glossy green leaves, which is typical of low-wax germplasm, compared to the WT, with *orp3a-21* being the most glossy. The lightness difference of WT plants was 4.3-, 5.8-, and 3.5-fold higher than in homozygous *orp3a-1*, *orp3a-21*, and *orp3a-34* mutants, respectively (Fig. 4c). These results indicate that the loss of BoORP3a function leads to a decrease in the cuticular wax contents of ornamental kale.

### Design and validation of a CAPS marker

**Figure 5:**
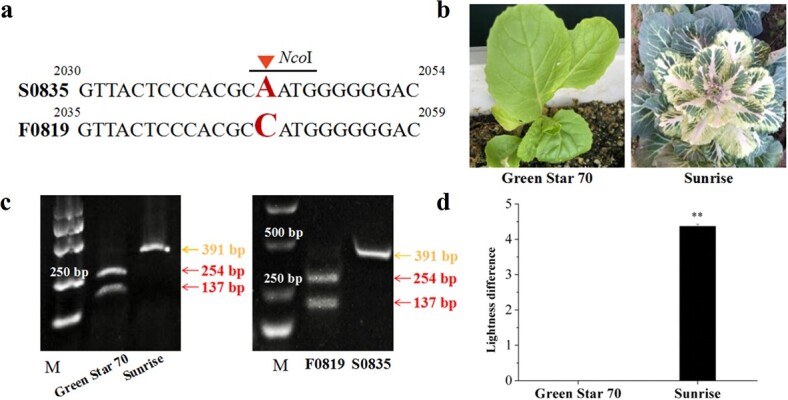
Validation of a CAPS marker associated with variation in wax deposition. **a** Partial sequence alignment of *BoORP3a* with the polymorphism between S0835 and F0819 (red letter) resulting in the introduction of a *Nco*I restriction site in the F0819 inbred line. **b** Representative images of Chinese cabbage ‘Green Star 70’ and ornamental kale ‘Sunrise’. **c** Results of PCR amplicons from Green Star 70, Sunrise, S0835, and F0819 after *Nco*I digest. Two bands indicate that the amplified products were digested. **d** Lightness difference for Green Star 70 and Sunrise. Data are means ± SD (*n* = 5). Asterisks indicate statistically significant differences (*P* < 0.01).

We designed a pair of primers to amplify a region of *BoORP3a* harboring a non-synonymous SNP between ornamental kale inbred lines S0835 and F0819. We then amplified genomic DNA from the two inbred lines, wax-free Chinese cabbage, and another ornamental kale with high wax (Sunrise). After enzymatic digestion with *Nco*I, agarose gel electrophoresis showed two bands (254 and 137 bp) for Chinese cabbage and the inbred line F0819, indicating the presence of the restriction site. By contrast, the PCR amplicons from the high-wax ornamental kale lines S0835 and Sunrise remained undigested (391 bp) (Fig. 5c). These results provide further evidence that *BoORP3a* is related to wax deposition.

## Discussion

Wax contents in plants are essential for hybridization breeding and commercial application. Recently, the accumulation of wax in *Brassica* vegetables has become a hot research topic [20, 21]. Ornamental kale is a popular vegetable and decorative plant whose cuticular wax contents affect its economic value. In this study, we established the presence of multiple SNPs in *BoORP3a* (*Bo9g184810*) between the high-wax inbred line S0835 and the low-wax line F0819. We knocked out *BoORP3a* in the high-wax line S0835 via genome editing, which resulted in lower cuticular wax contents in all three mutants isolated, as evidenced by colorimetry and the glossy phenotype of their leaf epidermis. Overall, our results demonstrate that the oxysterol-binding protein BoORP3a plays a role in the deposition of cuticular wax in ornamental kale.

Cuticular wax plays a critical role in protecting terrestrial plants against biotic and abiotic stresses [22]. The ultrastructure, composition, and contents of cuticular wax vary between species, organ type, developmental stage, and environmental conditions. Generally, in comparison to the old leaves, wax deposition was often lower in the young leaves in ornamental kale, which may be related to expression levels of wax biosynthetic genes, leaf positions and growth environments [23]. Some wax biosynthetic genes such as *BoLACS1.4* and *BoKCS1.1* were found to be highly expressed in young leaves, while *BoKCR1.1* and *BoCER3.4* were highly expressed in old leaves of cabbage [23]. In *A. thaliana*, *AtORP3a* (*AT5G02100*) showed a higher expression level in young leaves compared to old leaves [11]. In this study, we observed the structure of cuticular wax in the inbred lines S0835 and F0819 by SEM. We observed many irregular platelet-shaped wax crystals and a few tubular structures that were densely distributed on the leaf surface of ornamental kale plants, thus exhibiting slight differences with the cuticular wax crystals seen in cabbage [19]. Importantly, the contents, but not the morphology, of cuticular wax differed between the two inbred lines.

Many wax-deficient mutants have been described, most of them in Arabidopsis. Several of the causal genes have been identified and participate in wax biosynthesis, transport and export. For example, *CER2*, *CER3*, *CER6*, *CER10*, *3-KETOACYL-COA SYNTHASE1* (*KCS1*), *KCS2*, *BETA-KETOACYL REDUCTASE1* (*KCR1*), *PASTICCINO2* (*PAS2*), and *WSD1* are involved in wax biosynthesis [1]. CER5 and WHITE-BROWN COMPLEX HOMOLOG PROTEIN11 (WBC11) are ABC transporters and participate in wax transport with the lipid transfer protein LTPG [24–26]. In addition, MYB and APETALA2 (AP2)/ETHYLENE RESPONSE FACTOR (ERF) transcription factors have also been reported to directly or indirectly regulate wax biosynthesis [26, 27]. In contrast to wax biosynthesis, wax transport within the plasma membrane is not fully understood. Cuticular wax biosynthesis requires a large pool of lipids exported from epidermal cells to the plant surface [23]. The main two hypothetical mechanisms for transport of lipids from the ER to the plasma membrane are as follows: (i) Lipids move directly from the ER to the plasma membrane [28], and (ii) lipids travel to and through the Golgi apparatus from the ER and then move to the plasma membrane [29]. Therefore, the proteins involved in lipid transport between the ER, Golgi apparatus, and the plasma membrane may also relate to cuticular wax deposition.

Sterols play an essential part in plant growth and development and are a component of wax [4, 30]. Sterols are synthesized in the ER and are then rapidly transported via the Golgi to the plasma membrane [15]. OBPs and ORPs can bind various lipids and have multiple functions, such as sensing lipids and regulating cellular sterol distribution [13, 31]. For instance, PiORP1 may be involved in Pollen receptor-like kinase1 (PRK1) signaling during pollen development and growth in *Petunia inflata*, and this protein localizes to the plasma membrane of petunia pollen tubes [32]. However, little is known about the function of other plant ORPs. Arabidopsis ORP3a has been reported to cycle and transport sterols between the ER and the Golgi apparatus and might also transport other lipids [13, 33]. Therefore, we speculated that BoORP3a might function as a lipid transfer protein during wax transport. Unlike the gene mentioned above, the role of *BoORP3a* in wax deposition is largely unknown. Sequencing of *BoORP3a* from the two inbred lines revealed five amino acid substitutions in the low-wax line F0819 compared to the high-wax line S0835. ORP3a appeared relatively conserved among Brassicaceae. The Arabidopsis genome encodes 12 ORPs, while rice (*Oryza sativa*) contains six *ORP* genes; both families are also relatively conserved [11]. We determined that BoORP3a is located in the ER and the nucleus, which may indicate that ORP3a is an ER and nucleus-localized sterol-binding protein [13] and involves transporting sterols from ER to the PM. Similarly, ORP1S was identified to function as a cytoplasmic sterol sensor that transports sterols to the nucleus and facilitates sterol transfer between the ER and PM [34]. Overall, according to the localization of BoORP3a, it is speculated that BoORP3a may play a role in the process of lipid transfer from the ER to the PM, thereby affecting the wax transport.

CRISPR/Cas9-mediated genome editing has been instrumental in enabling improvements in several crop species. In recent years, it has been successfully used to improve *Brassica* vegetables, such as *Brassica rapa* [35] and *B. oleracea* [36]. The mutation efficiency varies depending on the species, the targeted gene, and the Cas9/sgRNA construct [37]. In this study, we applied CRISPR/Cas9 editing to knock out *BoORP3a* in an inbred line with high-wax contents, resulting in the identification of three independent mutants out of 40 primary transformants, corresponding to a transformation and editing efficiency of 10% and 7.5%, respectively. Importantly, *orp3a* mutants plants exhibited lower wax contents and had glossy leaves. We concluded from these results that BoORP3a might be associated with wax deposition in ornamental kale. These results suggest that CRISPR/Cas9-mediated editing is a promising and efficient technique for ornamental kale breeding programs. In addition, the CAPS marker designed based on a non-synonymous SNP site between the high wax and the low wax line could effectively distinguish the wax content trait. The results further demonstrate that *BoORP3a* is related to wax deposition, and this marker is also a promising molecular marker for marker-assisted selection in ornamental kale breeding.

In summary, we characterized the sequence of the candidate gene, *BoORP3a*, from high-wax (S0835) and low-wax (F0819) inbred lines and confirmed that this gene affects wax deposition. We identified six SNPs resulting in amino acid substitutions in BoORP3a between S0835 and F0819. BoORP3a is relatively well conserved in the Brassicaceae and localizes to the ER and the nucleus. *BoORP3a* knockout plants had lower cuticular wax contents and glossy leaves. However, how BoORP3a regulates the composition of the cuticular wax remains unclear and will be the focus of a future study.

## Materials and methods

### Plant materials and growth conditions

Seeds for the ornamental kale (*B. oleracea var. acephala*) high-wax (S0835) and low-wax (F0819) inbred lines (Fig. 1a) were obtained from the germplasm nursery of Shenyang Agricultural University and grown in a greenhouse in Shenyang, China, in 2020. The glossy and glaucous phenotypes could be observed visually at the rosette stage (about 2 months after sowing). At the rosette stage, 15 leaves of each inbred line were collected from five plants (three leaves per plant) for scanning electron microscopy (SEM) observations and determination of wax contents.

### SEM observations

Leaf samples were cut into about 3 × 5-mm pieces. After being fixed in specimen holders, each tissue fragment was frozen in liquid nitrogen and coated with gold particles in a preparation chamber. The wax deposition and distribution patterns were observed from the leaf surfaces of the two inbred lines with a scanning electron microscope (Regulus 8100, Hitachi, Japan).

### Determination of wax contents

The wax contents of leaf surfaces were evaluated with a colorimeter (CR-10 Plus, Konica Minolta, Japan). The L (lightness) index was used to evaluate the lightness change of the leaf surface as previously described [38]. The L values were measured before (L1) and after (L2) the gentle removal of cuticular wax and used to calculate the difference, ΔL = L1 – L2, which represents wax contents. Five biological replicates were examined for each genotype. The data in Figures 1b, 4c, and 5d are presented as means ± standard deviation (SD). SPSS software (version 22.0, SPSS Inc., Chicago, IL, USA) was used to perform a one-way analysis of variance (ANOVA) and LSD, with *P* < 0.05 considered a significant difference.

### Cloning and sequence analysis of *BoORP3a*

Genomic DNA extraction of all plant materials in this study was carried out using the cetyltrimethylammonium bromide (CTAB) method [39]. DNA integrity was analysed on 1% (w/v) agarose gels.

Primers specific for the *BoORP3a* gene (*Bo9g184810*) were designed according to the sequence obtained from the *B. oleracea* reference genome (http://plants.ensembl.org/Brassica_oleracea/Info/Index) (Table S1, see online supplementary material). The *BoORP3a* gene was then amplified from the DNA extracted above. The amplification conditions included a 3-min denaturation at 98°C, followed by 35 cycles of 95°C for 10 s, 50°C for 30 s, and 72°C for 2 min, then 72°C for 7 min and hold at 12°C. The PCR products were cloned into the pEASY-Blunt cloning vector (Transgen Biotech, China) and transformed into *Escherichia coli* strain DH5α. Positive transformants were sequenced by TSINGKE Biotechnology Company (Beijing, China). Each positive clone was sequenced at least twice.

The deduced amino acid sequences and conserved domains for BoORP3a were obtained from the NCBI database (https://www.ncbi.nlm.nih.gov/). Sequence alignment was performed with DNAMAN 6.0 software (Lynnon Biosoft, USA). Synteny information and homologous protein sequences of ORP3a among cruciferous species were obtained from the BRAD database (http://brassicadb.cn/) using BLAST and Syntenic Gene @ Subgenomes tools [40]. These sequences were used to construct a phylogenetic tree by the Maximum Likelihood method with the MEGA 7.0 software. The nonsynonymous substitution rate (Ka) and the HYPERLINK ''https://www.sciencedirect.com/topics/agricultural-and-biological-sciences/synonymous-substitution`` \o ``Learn more about synonymous substitution from ScienceDirect's AI-generated Topic Pages'' synonymous substitution rate (Ks) between paralogous gene pairs were calculated using the Kumar method implemented in MEGA 7.0 [41]. As a rule, neutral evolution is defined by a Ka/Ks value of one, a value higher than one indicates positive selection, while a value lower than one indicates purifying (negative) selection [42].

### Subcellular localization of BoORP3a

Total RNA was extracted using RNAiso reagent (TaKaRa Shuzo Co. Ltd, Japan) according to the manufacturer’s instructions. RNA integrity and purity were assessed on 1% (w/v) agarose gels with a NanoDrop 8000 spectrophotometer (Thermo Scientific, USA). First-strand cDNA synthesis was conducted with the AMV First Strand cDNA Synthesis Kit (Shanghai Sangon Biotechnology Co., Ltd).

The pCAMBIA1302-BoORP3a-eGFP expression vector was constructed using the subcellular localization primers to amplify the *BoORP3a* coding sequence (Table S1, see online supplementary material). The resulting plasmid was transformed into Agrobacterium (*Agrobacterium tumefaciens*) strain GV3101. Agrobacteria harboring the *35S:eGFP* or *35S:BoORP3a-eGFP* plasmids were then infiltrated into the leaves of 30-day-old *Nicotiana benthamiana* plants. An endoplasmic reticulum-specific marker (ER-rk) was co-infiltrated with the *BoORP3a-eGFP* or *eGFP* constructs. *N. benthamiana* plants were then cultivated in the dark for 18 h, followed by return to light for 24 h. GFP fluorescence was observed at an excitation wavelength of 488 nm and an emission wavelength of 510 nm with a confocal microscope (Leica TCS SP82400301, Germany) as described previously [43].

### Vector construction and genetic transformation

The knockout in *BoORP3a* was generated by CRISPR/Cas9-mediated genome editing as described previously [44]. Sequence-specific single guide RNAs (sgRNAs) were selected according to an online tool (http://crispor.tefor.net/), and the corresponding primers were synthesized (Table S1, see online supplementary material) and introduced into the pCBC-DT1T2 vector to produce a sgRNA expression cassette. The sgRNA expression cassette was cloned into the pHSE401 expression vector using the restriction enzyme *Bsa*I and T4 DNA Ligase. The resulting plasmid was introduced into Agrobacterium strain GV3101.

The genetic transformation of the high-wax line (S0835) was performed as described previously [45]. The S0835 seeds were sterilized and sown on Murashige and Skoog (MS) medium (pH 5.8) for 4–5 d and then cotyledons were cultivated in pre-cultivation medium [MS + 1.0 mg/L 6-benzylaminopurine (6-BA) + 0.1 mg/L 1-naphthaleneacetic acid (NAA) + 200 μmol/L acetosyringone (AS), pH = 5.8] for 2 d in the dark. The cotyledons were subsequently infected with Agrobacteria cell suspensions (OD_600_ = 0.8) for 5 min and then transferred to co-cultivation medium [MS + 1.0 mg/L 6-benzylaminopurine (6-BA) + 0.1 mg/L 1-naphthaleneacetic acid (NAA) + 200 μmol/L acetosyringone (AS), pH = 5.8] for 2 d. Afterwards, the transformed cotyledons were transferred to a delay culture medium (MS + 1.0 mg/L 6-BA +0.1 mg/L NAA + 300 mg/L cefotaxime, pH = 5.8) and selection medium (MS + 1.0 mg/L 6-BA +0.1 mg/L NAA +10 mg/L kanamycin +300 mg/L cefotaxime, pH = 5.8) in turn under a 16-h light/8-h dark photoperiod. After about 3 weeks, resistant buds were excised and transferred to new selection culture medium. When the resistant buds reached 2 cm in height, they were transferred to rooting culture medium (MS + 0.1 mg/L NAA + 15 mg/L kanamycin +300 mg/L cefotaxime, pH = 5.8) to obtain transgenic ornamental kale plants (Fig. S1, see online supplementary material).

The transgenic plants were confirmed by comparing gene sequences and phenotypes. Genomic DNA from individual transgenic plantlets was extracted by the CTAB protocol [39]. *ORP3a*-CRISPR-test-F/R-specific primers flanking the target sites sequence (Table S1, see online supplementary material) were designed to amplify a fragment of about 400 bp. The amplicons were cloned and sequenced as described above.

### Design of a Cleaved Amplified Polymorphic Sequence (CAPS) marker

To verify that *BoORP3a* is associated with cuticular wax deposition, a CAPS marker was designed based on the detected variation between the two inbred lines (Fig. 2c and 5a). The fragments were amplified with the primer pair CAPS-F, 5′-ACGTCTACGTCCTGATAGATAC-3′, and CAPS-R, 5′-TTAAGGAGAGGTATCTTGGAAC-3′ (annealing temperature 54°C). The amplicons were digested with *Nco*I enzyme for 30 min at 37°C before being separated on a 2% (w/v) agarose gel. The ornamental kale cultivar Sunrise (high-wax contents) and the Chinese cabbage (*B. rapa* spp. *pekinensis*) cultivar Green Star 70 (wax-free) were used to test the CAPS marker (Fig. 5b and d).

## Acknowledgements

This research was supported by the National Natural Science Foundation of China (32171850 and 31770739).

## Author contributions

S.Z.: vonceptualization, investigation, writing – original draft. F.Z. and Z.L.: conceptualization, investigation, formal analysis, writing – review and editing. X.F. and Y.L.: investigation, software. P.Z.: funding acquisition, project administration.

## Data availability

The data that support the results are included in this article and its supplementary materials. Other relevant materials are available from the corresponding author upon reasonable request.

## Conflict of interest

The authors declare that there is no conflict of interests regarding the publication of this article.

## Supplementary data

Supplementary data is available at *Horticulture Research* online.

## Supplementary Material

Web_Material_uhac219
